# Who Treats Patients with Diabetes and Compensated Cirrhosis

**DOI:** 10.1371/journal.pone.0165574

**Published:** 2016-10-26

**Authors:** Tsai-Ling Liu, A. Sidney Barritt IV, Morris Weinberger, John E. Paul, Bruce Fried, Justin G. Trogdon

**Affiliations:** 1 Department of Health Policy and Management, Gillings School of Global Public Health, University of North Carolina at Chapel Hill, Chapel Hill, NC, United States of America; 2 Center for Outcomes Research and Evaluation (CORE), Carolinas HealthCare System, Charlotte, NC, United States of America; 3 Division of Gastroenterology and Hepatology, University of North Carolina at Chapel Hill, Chapel Hill, NC, United States of America; 4 Durham VAMC Center for Health Services Research, Durham, NC, United States of America; University of Navarra School of Medicine and Center for Applied Medical Research (CIMA), SPAIN

## Abstract

**Background:**

Increasingly, patients with multiple chronic conditions are being managed in patient-centered medical homes (PCMH) that coordinate primary and specialty care. However, little is known about the types of providers treating complex patients with diabetes and compensated cirrhosis.

**Objective:**

We examined the mix of physician specialties who see patients dually-diagnosed with diabetes and compensated cirrhosis.

**Design:**

Retrospective cross-sectional study using 2000–2013 MarketScan^®^ Commercial Claims and Encounters and Medicare Supplemental Databases.

**Patients:**

We identified 22,516 adults (≥ 18 years) dually-diagnosed with diabetes and compensated cirrhosis. Patients with decompensated cirrhosis, HIV/AIDS, or liver transplantation prior to dual diagnosis were excluded.

**Main Measures:**

*Physician mix categories*: patients were assigned to one of four physician mix categories: primary care physicians (PCP) with no gastroenterologists (GI) or endocrinologists (ENDO); GI/ENDO with no PCP; PCP and GI/ENDO; and neither PCP nor GI/ENDO. *Health care utilization*: annual physician visits and health care expenditures were assessed by four physician mix categories.

**Key Results:**

Throughout the 14 years of study, 92% of patients visited PCPs (54% with GI/ENDO and 39% with no GI/ENDO). The percentage who visited PCPs without GI/ENDO decreased 22% (from 63% to 49%), while patients who also visited GI/ENDO increased 71% (from 25% to 42%).

**Conclusions:**

This is the first large nationally representative study to document the types of physicians seen by patients dually-diagnosed with diabetes and cirrhosis. A large proportion of these complex patients only visited PCPs, but there was a trend toward greater specialty care. The trend toward co-management by both PCPs and GI/ENDOs suggests that PCMH initiatives will be important for these complex patients. Documenting patterns of primary and specialty care is the first step toward improved care coordination.

## Introduction

Increasingly, patients with multiple chronic conditions are being managed in patient-centered medical homes (PCMH) [1,2] that seek to provide comprehensive, patient-centered, and coordinated care [3]. Central to the PCMH is coordinating primary and specialty care. Despite the increasing number of patients with multiple chronic conditions [4], little is known about the mix of PCPs and specialists treating patients other than cancer survivors. Before medical care can be coordinated, it is vital to understand the types of providers treating medically complex patients with multiple chronic diseases.

Cirrhosis is the 11^th^ leading cause of death in the United States [5], with the mortality rate increasing 40% in the past two decades [6]. Cirrhosis has two stages: compensated (patients with preserved liver function and no major complications) and decompensated (patients with major complications that require more intensive care). Because early cirrhosis is often asymptomatic, many patients are unaware of their disease [7] and, without proper care, are at risk for developing complications. A common comorbidity of cirrhosis is diabetes, which afflicts 28–40% of patients with compensated cirrhosis [8–10]. Patients dually-diagnosed with diabetes and compensated cirrhosis have a higher risk of developing decompensation events, including ascites, spontaneous bacterial peritonitis, variceal bleeding, hepatocellular carcinoma, and acute renal failure [10].

Patients with both diabetes and compensated cirrhosis may benefit from being managed by a mix of PCPs and specialists from gastroenterology and endocrinology. Several studies provide insights into the benefits of receiving care from both specialists and PCP among diabetes patients with chronic kidney disease [11,12], tuberculosis [13] and cancer [14–18]; however, little is known about patients dually-diagnosed with diabetes and compensated cirrhosis [19]. In addition, access to care may also influence on how these complex patients visited different physician specialty. Our study seeks to examine what physician specialties treat patients dually-diagnosed with diabetes and compensated cirrhosis between 2000 and 2013.

## Methods

### Data Source and Sample

**Fig 1:**
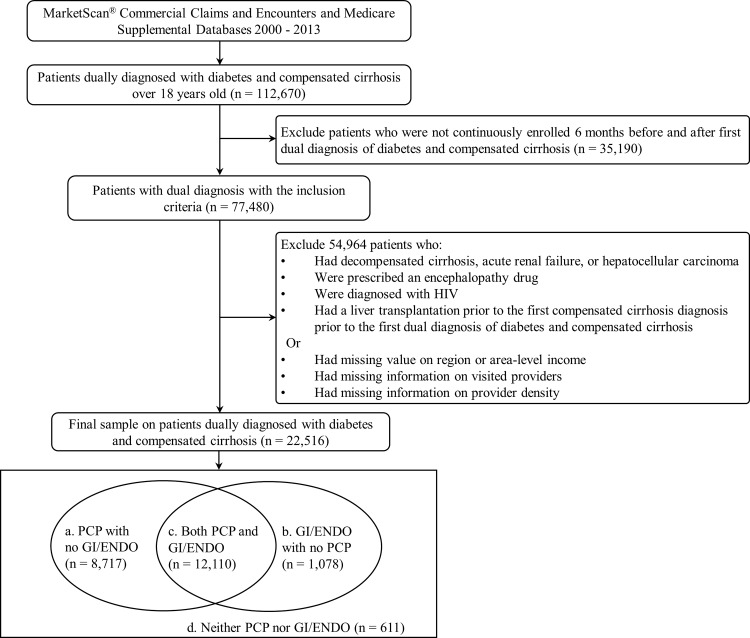
Patient flow for selecting dually diagnosed patients.

MarketScan^®^ Commercial Claims and Encounters and Medicare Supplemental Databases (Copyright 2015 Truven Health Analytics Inc. All Rights Reserved) between 2000 and 2013 were used for this retrospective cross-sectional study. Enrollees in MarketScan^®^ included employees insured by employer-sponsored plans and their dependents, as well as Medicare-eligible retirees with employer-provided Medicare Supplemental Plans. The sample included all patients 18 years of age or older who were enrolled for at least 6 months before and after the first date of being dual diagnosed with diabetes and compensated cirrhosis (Fig 1). The first dual diagnosis date of diabetes and compensated cirrhosis was defined as either the first date of diabetes after a diagnosis of compensated cirrhosis, or vice versa. Both diabetes and compensated cirrhosis were defined using the International Classification of Diseases, 9^th^ Revision, Clinical Modification (ICD-9-CM). Using data from the Outpatient Services Tables and Inpatient Admissions Tables, Diabetes was defined as either: 1) two or more different dates of service for a diabetic-related diagnosis (ICD-9-CM code: 250.xx) from the Outpatient Services Table or 2) at least one inpatient encounters with an ICD-9-CM code for diabetes [20]. Compensated cirrhosis was defined as alcoholic cirrhosis of the liver (ICD-9-CM code: 571.2), cirrhosis (ICD-9-CM code: 571.5), and biliary cirrhosis (ICD-9-CM code: 571.6) [21]. We excluded patients with decompensated cirrhosis (ICD-9-CM code: 789.59, 567.23, 456.00, 456.10, 456.2x, 572.20, 070.2x, 070.40, 070.44, 070.49, 070.60), acute renal failure (ICD-9-CM code: 584.xx), or hepatocellular carcinoma (ICD-9-CM code: 155.xx) diagnosed prior to the first dual diagnosis date. In addition, to avoid misclassification, patients who were prescribed an encephalopathy drug (Lactulose and Rifaxamin), had a diagnosis of HIV (ICD-9-CM code: 042.xx-044.xx), or had a liver transplantation (ICD-9-CM code: V42.7, ICD-9 procedure: 50.5, or CPT code: 47135, 47136) prior to the first dual diagnosis date were also excluded. The end of the study period was defined as the: 1) first drop-out date; 2) date of a serious complication (i.e. decompensation event, hepatocellular carcinoma, and acute renal failure); or 3) end of the study period (December 31, 2013). The study was exempted by University of North Carolina at Chapel Hill Institutional Review Board.

### Type of Physicians

Physicians who practiced in outpatient settings were identified using the “Provider Type” from the MarketScan Outpatient Services Table. Gastroenterologists (GI) and endocrinologists (ENDO) were defined as physicians in these specialties. Because there was no category for PCP, we used a definition used previously [14–17]: family practice, geriatric medicine, obstetrics/gynecology, internal medicine not elsewhere classified (NEC), medical doctor (NEC), and multi-specialty group practice. We categorized patients into four categories based on the physician mix visited: (1) PCP with no GI/ENDO, (2) GI/ENDO with no PCP, (3) both PCP and GI/ENDO, and (4) neither PCP nor GI/ENDO (Fig 1). We summed physician encounters by specialty in each year from 2000 to 2013.

### Physician Density

Previous studies have found that patients residing in higher physician density areas had better access to PCPs and specialists [22–24]. We focused on density of PCPs, GI/ENDOs, and other physicians. MarketScan contain enrollees’ five-digit Federal Information Processing Standard (FIPS) codes between year 2000 and 2010, but was dropped afterward due to privacy concerns. For patients who had their first dual-diagnosis of diabetes and compensated cirrhosis after 2011, metropolitan statistical area (MSA) was used to determine whether patients resided in a metropolitan area, as well as their state and county. Patients who resided in non-metropolitan areas after 2011 did not have county and state information and were dropped. The five-digit FIPS codes for 2001–2010 data, as well as state and county (linked through MSA) for 2011–2013 data, were identified with hospital referral regions (HRRs) through the Dartmouth Atlas Project (www.dartmouthatlas.org). The Dartmouth Atlas Project identified 306 HRRs based on where Medicare patients were admitted for tertiary care for major cardiovascular surgeries [25]. Density of PCP, GI, ENDO, and other specialties per 100,000 residents in each HRR were only provided in 1996, 2006, and 2011. We linked the physician density with the closest year: 2000 and 2001 were linked with physician density in 1996; 2002–2008 with physician density in 2006; and 2009–2013 with physician density in 2011. If multiple HRRs were linked to single patient, weighted physician densities based on the total population in each HRR were calculated.

### Health Care Utilization

Annual physician visits and annual health care expenditures for each patient were used as indicators of health care utilization. Both variables came from the MarketScan Outpatient Services Table. Physician visits were summed for each physician mix category during the observation period. Total health care expenditures were the sum of deductible, coinsurance, coordination of benefits and other savings, and the net payments from each outpatient visit. In addition, total health care expenditures were adjusted by inflation using the Medical Care Consumer Price Index in 2013 dollars. Average physician visits and health care expenditures per patient per year were reported.

### Covariates

Demographic variables, including age, gender, geographic region, and urban/rural residence were identified through the Annual Enrollment Summary Table. The Elixhauser Comorbidities index [26,27] was defined for the six months prior to the first dual diagnosis date. To avoid collinearity, diseases related to liver disease and diabetes were excluded when calculating the Elixhauser Comorbidity Index; the remaining 28 comorbidities were summed (0–28). Because socioeconomic status (SES) was not available in the database, area-level median income was used as a proxy. Area-level median income provided by the Small Area Estimates Branch, U.S. Census Bureau was linked through five-digit FIPS code between 2000 and 2010 and MSA code between 2011 and 2013.

### Data Analysis

Our analysis focused on investigating the characteristics that affect dually-diagnosed patients’ use of physician mix categories. We first examined patient characteristics, the distribution of visits to physician mix categories, and the number of physician encounters between 2000 and 2013. Patient encounters were analyzed separately for additional information. We then compared the percentage and average number of annual visits to each physician specialty by physician mix category. Time trends for the percentage and the number of visits for physician mix categories were analyzed. Furthermore, to understand the characteristics that affect patients’ physician mix category, a multinomial probit model was estimated to compare the odds of visiting different physician mix categories, controlling for age, gender, geographic location, urban/rural residence, physician density, number of comorbidities, and area-level median income. Marginal effects on the probability of visiting each physician mix category and confidence intervals (CI) were calculated and reported based on the delta method. A p-value < 0.05 was considered statistically significant. All analyses were conducted using SAS for Windows, Version 9.4 (SAS Institute Inc, Cary, NC, USA) and STATA 14.0 (STATA Corp, College Station, TX, USA).

## Results

The 22,516 patients (47,985 patient-years) in the final sample (Fig 1) had 1,151,542 encounters during the 14-year study period. Approximately half of patients (54.0%) were male, 25.6% were over 65 years of age, 86.4% resided in urban areas; patients had a mean of 1.88 comorbidities besides diabetes and cirrhosis (Table 1). In addition, patients had a mean of 18.7 months of observation time (median of 11.2 months). Patients who visited GI/ENDO with no PCP had the fewest comorbidities; patients who visited PCPs, with or without GI/ENDO, had the most comorbidities.

**Table 1:** Descriptive distribution of patients who were dually diagnosed with compensated cirrhosis and diabetes by physician mix category, MarketScan 2000–2013.

	Total (n = 22,516)	PCP with no GI/ENDO (n = 8,717)	GI/ENDO with no PCP (n = 1,078)	PCP and GI/ENDO (n = 12,110)	Other physician (n = 611)
Total dual diagnosed patients (%)		38.7	4.8	53.8	2.7
Female (%)	46.0	43.3	38.2	49.0	39.8
Age group (%)					
Under 40	3.5	3.9	4.3	3.1	3.6
40–44	4.6	4.9	4.4	4.3	4.4
45–49	9.3	9.5	10.9	9.0	10.8
50–54	16.6	15.2	16.4	17.7	14.7
55–59	21.6	19.4	22.2	23.2	20.0
60–64	18.8	18.7	21.4	18.5	23.1
65+	25.6	28.5	20.4	24.1	23.4
Region (%)					
Northeast	13.9	12.3	17.4	14.7	14.7
Midwest	28.3	32.8	18.1	26.4	21.4
South	51.5	47.1	60.2	53.6	58.4
West	6.2	7.7	4.3	5.3	5.4
Rural area (%)		38.7	4.8	53.8	2.7
Urban area (%)	46.0	43.3	38.2	49.0	39.8
Elixhauser Comorbidity Index (mean ± SD)	1.9 ± 1.7	2.0 ± 1.8	1.7 ± 1.6	1.8 ± 1.7	2.2 ± 1.8
Median income in 10K (mean ± SD)	5.1 ± 1.3	5.0 ± 1.2	5.2 ± 1.3	5.1 ± 1.3	5.0 ± 1.2
Observed length in months (mean ± SD)	18.7 ± 21.5	16.2 ± 19.7	5.5 ± 7.7	22.3 ± 23.0	5.4 ± 8.6
Number of physician visits (mean ± SD)	51.1 ± 77.4	41.7 ± 67.3	12.2 ± 33.0	63.6 ± 85.4	8.9 ± 23.2
Healthcare expenditure in 1K^*^ (mean ± SD)	4.2 ± 15.9	3.3 ± 9.0	1.1 ± 2.8	5.2 ± 20.2	1.1 ± 4.4

*Healthcare expenditures were adjusted by inflation using the Medical Care Consumer Price Index in 2013 dollars.

**Fig 2:**
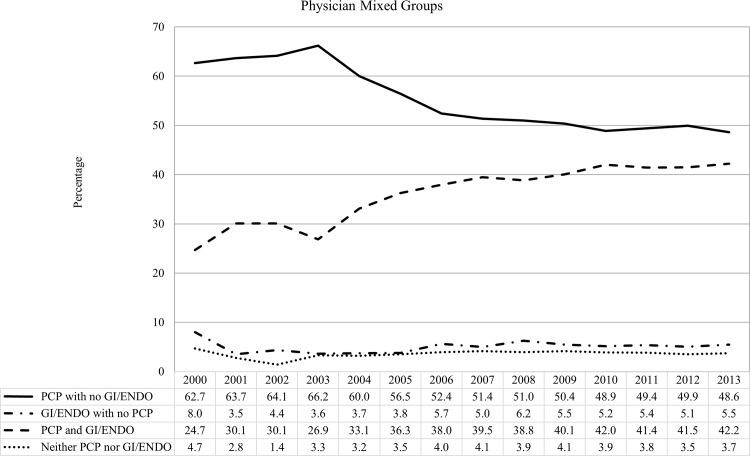
Distribution of physician mix categories among dually diagnosed patients.

During the 14-year study, 92.5% of patients visited a PCP (53.8% visited PCP and GI/ENDO and 38.7% visited PCP with no GI/ENDO) and 58.6% visited any GI/ENDO (53.8% visited PCP/GI/ENDO and 4.8% visited GI/ENDO with no PCP). In addition, 2.7% of patients did not visit any PCP, GI, and/or ENDO. Between 2003 and 2006, the distribution of physician mix changed from visiting PCP only to both PCPs and specialists (Fig 2). Overall, the number of patients who visited both PCPs and specialists (GI and ENDO) increased more than 70% between 2000 (24.7%) and 2013 (42.2%). About 4% of patients in any given year did not visit either PCP or GI/ENDO, but the percentage decreased 21% from 2000 to 2013. Overall, a large proportion of patients visited PCPs only in any given year throughout the 14 years of observation.

At the encounter-level, 57.1% of all encounters were with PCPs, 5.9% of encounters were with GIs, and only 3.0% of encounters were with ENDOs. Other provider encounters included cardiovascular disease specialists (6.4%), oncology (4.7%), and ophthalmology (3.4%). The remaining provider encounters included a diverse group of specialists, each representing less 3% of all encounters (Table 2).

**Table 2:** Distribution of total physician encounters by specialties, 2000–2013.

	n	%
Total	1,151,542	
Primary Care Physician	657,604	57.1
Cardiovascular Dis/Cardiology	73,235	6.4
Gastroenterology	68,370	5.9
Oncology	54,480	4.7
Ophthalmology	38,715	3.4
Endocrinology & Metabolism	35,196	3.1
Hematology	30,887	2.7
Emergency Medicine	28,512	2.5
Dermatology	22,517	2.0
Urology	21,299	1.8
Rheumatology	16,158	1.4
Pulmonary Disease	15,111	1.3
Neurology	13,393	1.2
Nephrology	13,326	1.2
Physical Medicine & Rehab	12,842	1.1
Otolaryngology	11,612	1.0
Psychiatry	10,271	0.9
Infectious Disease	8,216	0.7
Allergy & Immunology	6,976	0.6
Hospitalist	2,923	0.3
Pediatrician (NEC)	2,296	0.2
Critical Care Medicine	2,209	0.2
Plastic/Maxillofacial Surgery	2,176	0.2
Osteopathic Medicine	1,994	0.2
Preventative Medicine	354	0.0
Proctology	263	0.0
Pediatric Specialist (NEC)	183	0.0
Pediatric Orthopaedics	178	0.0
Neonatal-Perinatal Medicine	105	0.0
Sports Medicine (Pediatrics)	59	0.0
Palliative Medicine	45	0.0
Genetics	34	0.0
Pediatric Urology	3	0.0

**Fig 3:**
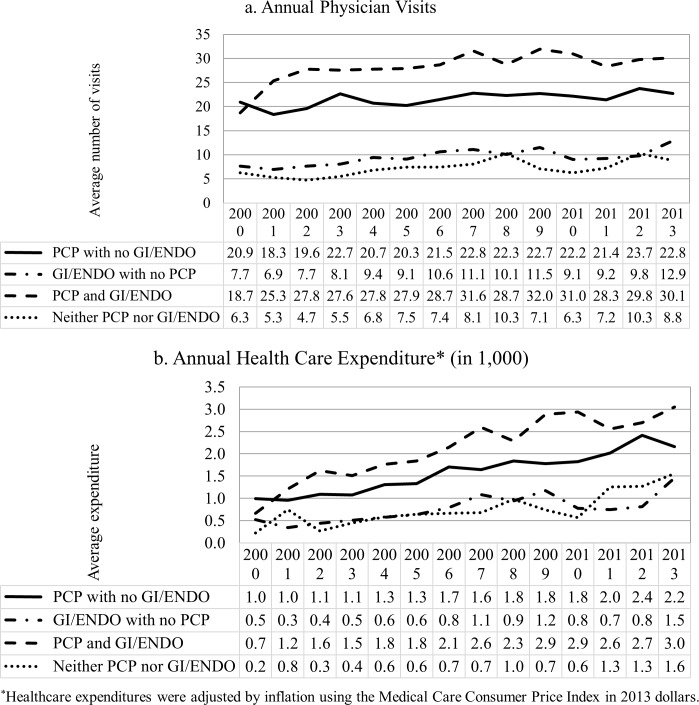
Average number of annual health care utilization among dually diagnosed patients, by physician mix category.

**Fig 4:**
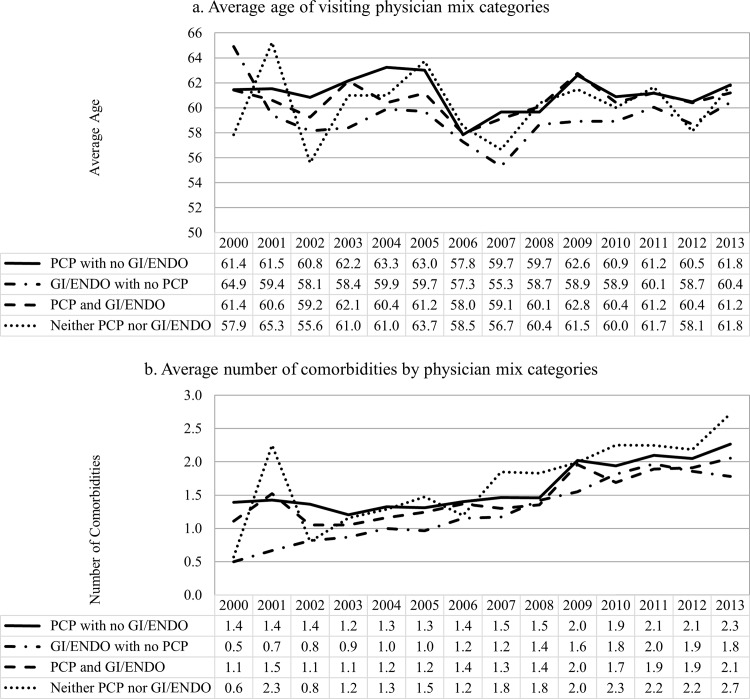
Average age and number of comorbidities by physician mix categories by year.

The trend of annual physician visits was very similar across physician mix categories (Fig 3). On average, patients who visited both PCP and GI/ENDO had the highest number of total physician visits, followed by patients who visited PCP with no GI/ENDO. The same pattern can be found in total health expenditures, but the health care expenditure increased steadily over the past decade. Patients who visited PCPs only had the fewest comorbidities; while the number of comorbidities among patients who visited both PCP and GI/ENDO were only slightly higher than the PCP only group (Fig 4). The average number of comorbidities steadily increased after 2002.

**Fig 5:**
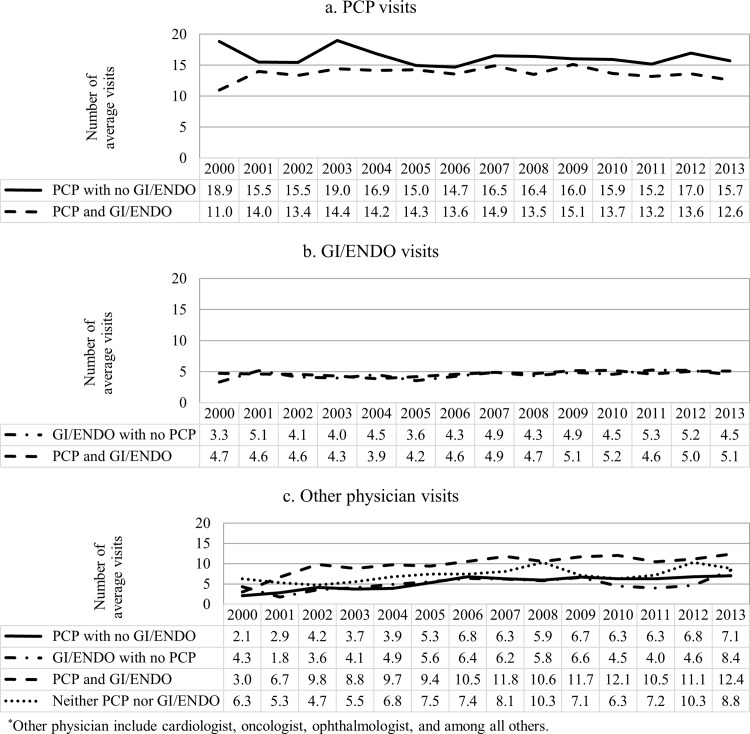
Average number of annual visits to physician specialties among dually diagnosed patients, by physician mix category.

Patients had a mean of 15.0 visits to any PCP, 4.4 visits to any GI/ENDO, and 7.2 visits to any other physicians throughout the study period (Fig 5). Patients who were in PCP/GI/ENDO group had slightly lower mean number of PCP visits than the PCP with no GI/ENDO group in every year of estimate, but had the highest mean number of visits to other physician than the rest of the physician mix categories. Among patients who had visited any GI/ENDO, the mean number of visits was similar between those with and without a PCP visit.

After adjusting for patient characteristics and physician density, male patients, patients with a higher number of comorbidities and lower median incomes had a higher probability of visiting PCPs with no GI/ENDO; while female patients, those with fewer comorbidities, and those with higher median incomes had higher probability of visiting the specialist with or without PCP (Table 3). Moreover, patients who resided in higher PCP density areas tend to visit PCP more than any other specialties, while patients who resided in higher GI/ENDO density area had higher probability of visiting any GI/ENDO and lower probability of visiting any PCP. Finally, patients who resided in rural areas had statistically significant lower probability of visiting any GI/ENDO specialist.

**Table 3:** Marginal effects on probability and 95% confidence interval of visiting different physician mix categories using multinomial probit model.

	PCP with no GI/ENDO	GI/ENDO with no PCP	PCP and GI/ENDO	Neither PCP nor GI/ENDO
Physician density (per 100K)				
PCP	0.0009***	-0.0002*	-0.0006*	-0.0001
GI/ENDO	-0.0269***	0.0071**	0.0163**	0.0036*
Neither PCP nor GI/ENDO	0.0015***	-0.0001	-0.0013***	-0.0001
Female	-0.0474***	-0.0146***	0.0692***	-0.0072**
Age group				
Under 40				
40–44	-0.0176	-0.0121	0.0325	-0.0028
45–49	-0.0431*	-0.0028	0.0440*	0.0019
50–54	-0.0858***	-0.0098	0.1004***	-0.0048
55–59	-0.0940***	-0.0078	0.1058***	-0.0039
60–64	-0.0590**	-0.0011	0.0566**	0.0036
65+	-0.0228	-0.0152	0.0429*	-0.0048
Region				
Northeast				
Midwest	0.0605***	-0.0137*	-0.0419**	-0.0050
South	-0.0168	0.0096	0.0042	0.0030
West	0.0936***	-0.0105	-0.0818***	-0.0013
Rural area	0.0108	-0.0146**	0.0072	-0.0034
Number of comorbidities	0.0132***	-0.0032***	-0.0131***	0.0031***
Median income in 10K	-0.0188***	0.0017	0.0191***	-0.0020

*P < 0.05

**P < 0.01

***P < 0.001

## Discussion

This is the first national study to examine patterns of outpatient care for patients with dually-diagnosed diabetes and compensated cirrhosis, a group with high and costly health care utilization. These complex patients may be best managed by both outpatient PCPs and specialist physicians. Previous studies have shown that the role of PCPs in managing patients with compensated cirrhosis was to identify risk factors, improve quality and length of life, and prevent patients from complications [28]. Specialists traditionally treat the complications and select patient candidates for liver transplantation when necessary [28]. One study found that patients had better outcomes when managed by both PCPs and GIs when admitted to hospital for a decompensation event [29]. Another study found that local access to subspecialty care increases the chance of patients receiving a liver transplant [24]. However, we are not aware of any studies investigating the mix of physician specialties treating patients with diabetes and compensated cirrhosis.

We found that more than 90% of these patients visited PCPs; 39% of all patients only visited PCPs. When examining trends in the mix of physician visits, more than half of our sample visited PCPs but not any specialists each year. Notably, the percentage of patients visiting a PCP decreased 22% between 2000 (63%) and 2013 (49%), while the share of patients who visited both PCPs and specialists increased by over 70% during the same period (from 24.7% in 2000 to 42.2% in 2013). One explanation for this shift may be the increasing emphasis on the PCMH: if patients with diabetes are diagnosed with compensated cirrhosis are referred to the GI, it could explain the increasing percentage of patients visiting both PCPs and GIs. As these patients increasingly visit both PCPs and specialists, the PCMH becomes critical to coordinate care. Several studies have found that the PCMH model was able to successfully reduce cost and ED utilization only among patients with complex chronic conditions [30,31]. Therefore, the PCMH and other coordinated care models may be especially critical for complex patients such as those in our study. Other events that temporally coincide with an increase in subspecialty care around 2002–3 are the changes to the model for end stage liver disease for liver transplant organ allocation [32] and the use of pegylated interferon and ribavirin for the treatment of hepatitis C [33]. Hence, patients with compensated cirrhosis were more likely to be referred to GI from PCP after the treatment pattern changes.

The mean number of visits to PCPs was about ten times higher than to GI/ENDO. However, the mix of physicians treating patients in our sample is very different from the mix of physicians found in studies of breast cancer [16] and colorectal cancer [14,15] survivors with comorbid chronic diseases. About one quarter of breast cancer survivors visited a PCP, but not an oncologist, while more than half visited both PCPs and oncologists [16]. Colorectal cancer survivors tended to visit PCPs but not oncologists, although visiting both PCPs and oncologists remained the second largest group [14,15]. Our study found that during the 14-year study period, patients dually-diagnosed with diabetes and compensated cirrhosis commonly visited both PCP and specialists, but the distribution changed over time with increased visits to both PCP and GI/ENDO.

Visiting a mix of physician specialties was correlated with increased age, female gender, physician density, number of comorbidities, and median income in our study. Although patients who visited a GI/ENDO with no PCP seem to have the least number of comorbidities in any given year, they were relatively younger than patients in other physician mix categories. On the other hand, patients who visited both PCP and GI/ENDO had the highest total visits and total expenditures, but their mean number of comorbidities was almost the same as those who visited PCP only. Patients who lived in rural areas were more likely to see a PCP, while those residing in urban areas were more likely to see a specialist only. In addition, to take enrollment months into account, we conducted the sensitivity analysis by including observed lengths in months as a covariate in the multinomial probit model. The length of enrollment was positively associated with the probability of seeing both a PCP and GI/ENDO, but the inclusion of the variable had little qualitatively impact on the other coefficients in the model. If coordinated care between specialist and PCPs does indeed improve outcomes, geographic access to care will be an important area for improvement in this population.

Our study had some limitations. First, our sample included only persons who were enrolled in employer-sponsored plans and/or Medicare Supplemental plans; therefore, our findings may not generalize to persons who are uninsured or insured with other types of programs. Second, some patients were dropped due to incomplete physician density because of the data structure and data linkage. This was because we can only access three years of physician density from Dartmouth Atlas of Health Care, and these patients were unable to link the corresponding physician density through FIPS and MSA. Third, we lacked county and state information for patients diagnosed after 2011 and resided in non-metropolitan areas. However, we tried to obtain the most relevant physician density each year by linking the physician density with the closest time period. Finally, MarketScan data lack information about important patient characteristics that affect access to care, for example, SES and race/ethnicity. Thus, we were unable to consider these variables in our analyses. However, we used area-level median income as a proxy to estimate SES.

The prevalence of and diabetes [34] and cirrhosis [7] alone, and in combination [35,36] is increasing, as is the morbidity, suffering, and health care costs these patients face. By understanding outpatient visit patterns among these patients, we can develop appropriate strategies to efficiently manage and improve their health. We found that while the proportion of patients who visited both PCPs and GI/ENDOs increased dramatically in the past decade, PCPs still carry much of the burden of caring for these complex patients. With the proliferation of the PCMH, coordinating PCPs and specialists care will be critical. Future research is needed to determine whether patients with diabetes and compensated cirrhosis could similarly benefit from coordinated care from PCMHs that involve both PCPs and specialists.
