# Correction: Who Treats Patients with Diabetes and Compensated Cirrhosis

**DOI:** 10.1371/journal.pone.0190756

**Published:** 2018-01-02

**Authors:** Tsai-Ling Liu, A. Sidney Barritt, Morris Weinberger, John E. Paul, Bruce Fried, Justin G. Trogdon

There is an error in the XML that is causing the second author’s name, A. Sidney Barritt 4^th^, to be indexed incorrectly. The name should be indexed as Barritt AS 4^th^ and not Barritt IV AS. The correct citation is: Liu T-L, Barritt AS 4^th^, Weinberger M, Paul JE, Fried B, Trogdon JG (2016) Who Treats Patients with Diabetes and Compensated Cirrhosis. PLoS ONE11(10): e0165574. https://doi.org/10.1371/journal.pone.0165574
